# Flexibly actuated pneumatic extrusion with in-situ monitoring for direct ink writing of heterogeneous and pressure-vulnerable materials

**DOI:** 10.1038/s41598-025-15164-9

**Published:** 2025-08-19

**Authors:** Chaiwuth Sithiwichankit, Setthibhak Suthithanakom, Kantawatchr Chaiprabha, Kai Melde, Ratchatin Chancharoen

**Affiliations:** 1 Department of Mechanical Engineering, Faculty of Engineering, Chulalongkorn University, Bangkok, Thailand; 2 Institute for Molecular Systems Engineering and Advanced Materials, Heidelberg University, Heidelberg, Germany; 3 Human-Robot Collaboration and Systems Integration Research Unit, Chulalongkorn University, Bangkok, Thailand

**Keywords:** Direct ink writing, Material extrusion, Bioprinting, Flexible actuation, In-situ monitoring, Biomedical engineering, Mechanical engineering, Biomaterials, Soft materials

## Abstract

This study presents a novel piston-driven pneumatic extrusion system for direct ink writing (DIW), featuring flexible actuation and real-time monitoring of extrusion pressure. The design integrates the benefits of both pressure and feedrate control, achieving consistent linewidth while safeguarding pressure-sensitive materials such as cell-laden hydrogels. The system comprises a lightweight pneumatic syringe on the printhead and a stationary actuation unit, allowing efficient decoupling of motion and extrusion. Experiments demonstrate stable gelatin extrusion with a mean linewidth of 4.32 mm and a minimal increase ratio of 0.012 over printing distance. These findings show promise for advancing DIW with emerging soft materials, particularly in bioprinting and sustainable manufacturing.

## Introduction

Liquid deposition modeling, commonly called direct ink writing (DIW), is an extrusion-based additive manufacturing technique used for processing liquid or paste-like materials^1–3^. This technology exhibits extraordinary features of customization and rapid prototyping such that it can fabricate intricate structures that may be challenging or impossible to achieve with traditional manufacturing methods^4^. As a versatile tool, DIW is applicable for use with diverse materials, including soft polymers^5,6^biomaterials^7^ceramics^8,9^metals^10^and composites^3,11^. DIW is employed in extensive fields (bioengineering^12^food technology^13,14^and flexible electronics^15,16^, fostering innovation in a broad range of manufacturing and research^17,18^.

In bioprinting, DIW facilitates the fabrication of intricate 3D biostructures^19^ and numerous types of tissue, such as skin^20^bone^21^and vascular^22^to meet clinical requirements^23^. Thus, DIW offers customization and solutions for personalized treatments: namely, tailored drug delivery^12^ and transplantation^24^. In the food industry, DIW has revolutionized food products, offering opportunities for personalized and healthy eating solutions^25,26^. High quality products manufactured through this process help reduce overproduction and waste, aligning with the principle of a circular economy^27,28^. Moreover, DIW has played a crucial role in flexible electronics^29^. Using conductive soft materials, this technique enables the manufacturing of various compliant electronic components, such as actuators^30^sensors^15^and energy storages^31^. This revolution has led to significant advancements in wearable devices^32^.

In the DIW process, the steadiness of material flow greatly impacts the quality of material deposition and printing linewidth, affecting the success of fabrication^33,34^. The modeling and control of material flow is an important factor in the process, aligning with the mainstream research in this field^35,36^. This issue is influenced by the rheological properties of a printing material and the specifications of a material extruder^37–39^. Materials used in DIW extensively exhibit viscoelasticity^40^performing energy storing and dissipation^41^. Under slightly different conditions, such as temperature, pressure, and flow rate, the rheological properties of viscoelastic materials can be extremely diverse^42,43^. Material viscoelasticity leads to intricate characteristics of material flow^44,45^. During the printing process, the complex internal structure of a material extruder greatly impacts material flow.

In the printing of nonhomogeneous and delicate materials like cell-laden hydrogels and other biomaterials, rheological properties of such materials can be undetermined and vary over a wide range^46,47^, A small change in temperature can have dramatic effects on their rheological properties. This challenge is further amplified by the growing adoption of multi-material printing, which expands the diversity of printing materials. In fact, hydrogels are printable only in their phase transitioning between solution and gel^48^. These materials are unable to hold their shapes against gravity in solution state, and have remarkable flow resistance in gel state. During state transition, the materials exhibit distinctive heterogeneous properties. In addition, under excessive pressure, biological cells and their molecular structures can be damaged by being subjected to overloaded shear stress, reducing their viability^49^. Printing resolution is inherently affected by cell size as larger nozzles are required to prevent clogging, and minimize harm on cells. To obtain consistent printing linewidth of biomaterials, feedrate control is favourable. Meanwhile, extrusion pressure must be monitored to protect cells and biochemicals, from excessive pressure. Once the pressure is beyond designated thresholds, pressure control should override the extrusion process for protection. Using both pressure and feedrate control in extrusion is therefore beneficial for this application^50^.

A review on the landscape of DIW extrusion suggests that there are three traditional methods: pneumatic, piston, and screw extrusion^51^. These methods rely on two different principles of actuation. The first one is pressure control, and the other one is feedrate control. In pressure control, extrusion pressure is handled, and the flow of printing materials depends on their rheological properties and the pressure^52^. The visibility of extrusion pressure is valid, but the roughness of linewidth cannot be assured according to the possibility of the variation in material properties. On the other hand, feedrate control operates on a volumetric basis^53^. Extrusion feedrate is managed under motion control, inherently enabling a steady linewidth, despite variations in material properties. However, this concept of extrusion generally lacks the capability of sensing extrusion pressure, which tends to significantly fluctuate during extrusion. In addition, these two actuation principles cannot be activated simultaneously.

**Fig. 1:**
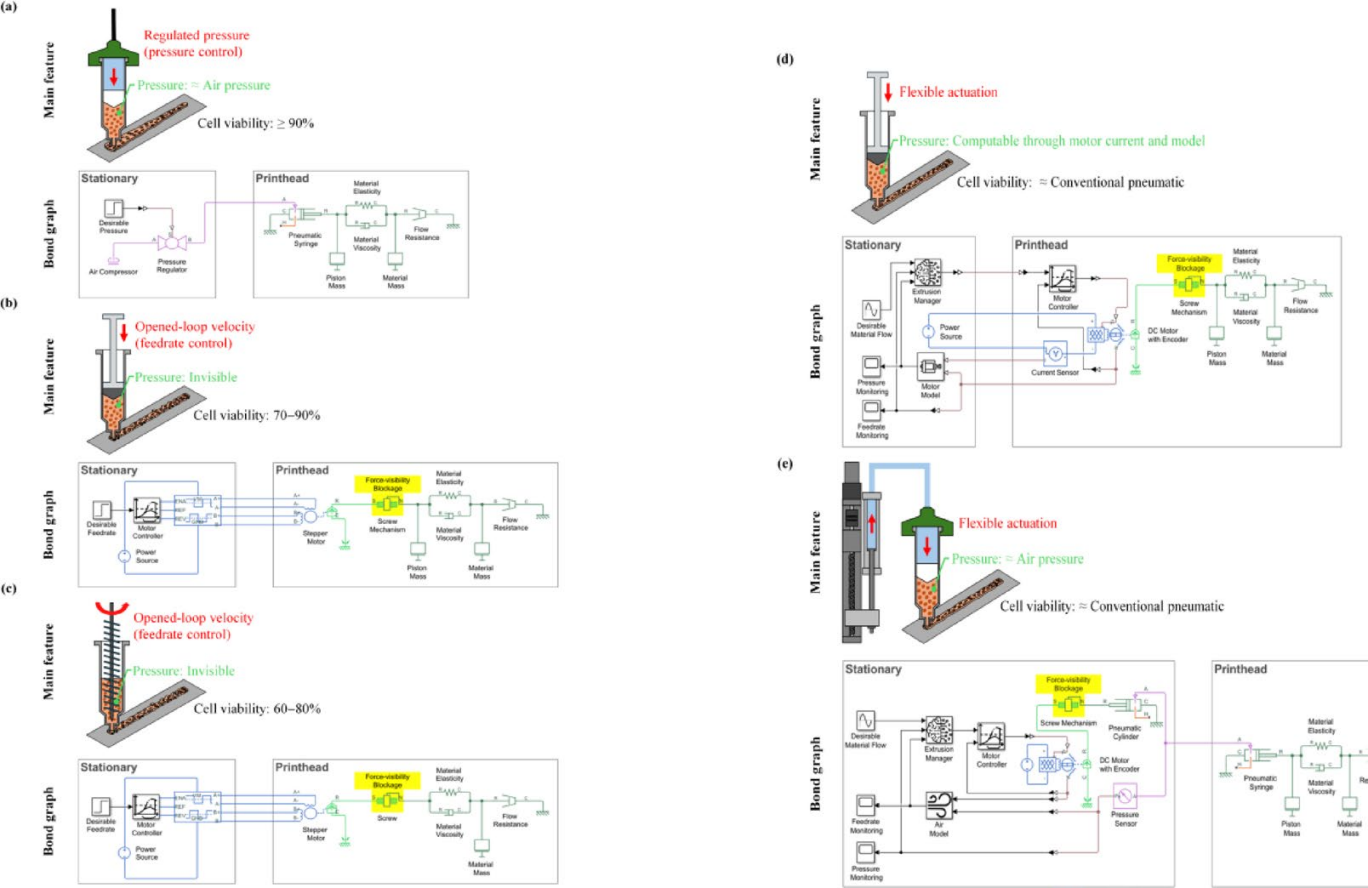
DIW extrusion of cell-laden hydrogels with bond-graph representations of various extrusion systems: (**a**) Conventional pneumatic extrusion^55,56^(**b**) Conventional piston extrusion^59,60^(**c**) Conventional screw extrusion^61^(**d**) Flexibly actuated piston extrusion with DC motor and encoder, and (**e**) Flexibly actuated piston-driven pneumatic extrusion. DIW extrusion of cell-laden hydrogels with bond-graph representations of various extrusion systems: (**a**) Conventional pneumatic extrusion^55,56^(**b**) Conventional piston extrusion^59,60^(**c**) Conventional screw extrusion^61^(**d**) Flexibly actuated piston extrusion with DC motor and encoder, and (**e**) Flexibly actuated piston-driven pneumatic extrusion.

Pneumatic extrusion is typically implemented following pressure control^54^. By exploiting pressurized air, actuation is accomplished. Air pressure is monitored and regulated in real-time (Fig. 1a). Because extrusion pressure is literally equivalent to air pressure, it is recognizable throughout the process. While other parts can be stationed on the ground, a pneumatic syringe is the only essential component on the printhead in this method. This configuration allows printheads to be as compact and lightweight as possible. Besides, a single actuation system can work with various dimensions of pneumatic syringes. It has been revealed that cell viability of bioprinting can reach over 90%, using this extrusion technique^55,56^.

In piston^57^ and screw^58^ extrusion, feedrate control is utilized (Fig. 1b and c). Actuation is commonly powered by stepper motors, driving pistons and screws without feedback compensation. If the motors can maintain their stepping, the pistons and screws are propelled and rotate reliably, giving stable printing linewidth, at steady state. Due to the need of a motor and mechanical transmission, the printheads in both piston and screw extrusion systems are likely to be large and heavy. Mechanical transmission in these extrusion types limits the options available for syringe and screw sizes, and geometries. Several studies report that cell viability is around 70–90% for piston extrusion^59,60^and 60–80% for screw extrusion^61^.

In the aspect of hygiene, syringes are the only components that directly contact printing materials in pneumatic and piston extrusion. Syringes are designed to be modular, so they can be disposed of or sterilized conveniently to prevent contamination. Unlike pneumatic and piston extruders, screw extruders must be disassembled before being sanitized. In contrast, screw extrusion is the only method that is conventionally viable for the DIW process at high temperature.

Previously, we proposed the concept of flexible actuation where material extrusion is managed, utilizing both control principles and event-driven switching techniques (Fig. 1d)^62^. This concept was implemented using a piston extruder driven by a DC motor with an encoder. In this configuration, extrusion pressure is estimated by monitoring the motor current, which is converted into pressure employing a transmission model. Even though pressure is visible in this design, when motor power is disabled, pressure visibility is blocked^63^. Friction in the screw transmission causes fluctuations in mechanical advantage, which in turn affects pressure sensing. Given the current limitations, further research into material extrusion in DIW is motivated.

In this study, flexibly actuated material extrusion with direct visibility of extrusion pressure for DIW is proposed (Fig. 1e). This idea is proceeded with a piston-driven pneumatic system, where pressure can be inherently measured through the air pressure in the pneumatic connection. During extrusion, the pneumatic connection is enclosed, allowing the extrusion feedrate to be calculated from the volume of the working air, which can be determined using an air model. Without supplementary sensing components that directly contact the printing materials, contamination is practically avoidable. In high-temperature operations, where a pneumatic syringe performs without piston, enhancement in printing quality is also provided. Except for the syringe mounted on the printhead, all actuation components are placed on the base. The increase in printhead mobility ideally improves both printing continuity and quality. Furthermore, the mechanical decoupling between the syringe and stationary actuation system offers flexible customization of syringe capacity and shape. Pneumatic transmission corresponds to pressure advance in fused deposition modeling, where the dynamics of filaments is compensated by a predetermined model^64^. Despite the increase in printhead mobility, this concept requires superior computation power to handle air dynamics and manage extrusion feedrate. To the best of our knowledge, no prior report has clearly described flexible material extrusion alongside native and persistent sensing on extrusion pressure, especially without additional contact sensors. Herein, our work is new and authentic.

This research advances the extrusion process in DIW, specifically for pressure-vulnerable materials with nonhomogeneous properties. While consistent material extrusion is a crucial precondition in DIW fabrication, this advancement remarkably impacts the fabrication of such materials, expanding the range of printable designs and applications, especially in bioprinting. Its findings contribute to bioengineering and other related fields. At a broader scale, the research contributions align with real-world problems and progress towards the sustainable development goals^65^. A major limitation of this work is that it focuses on the steady state of material extrusion. For satisfactory transient material flow, Lopez-Donaire et al. highlight that preliminary knowledge of the printing materials is essential^66^.

**Fig. 2:**
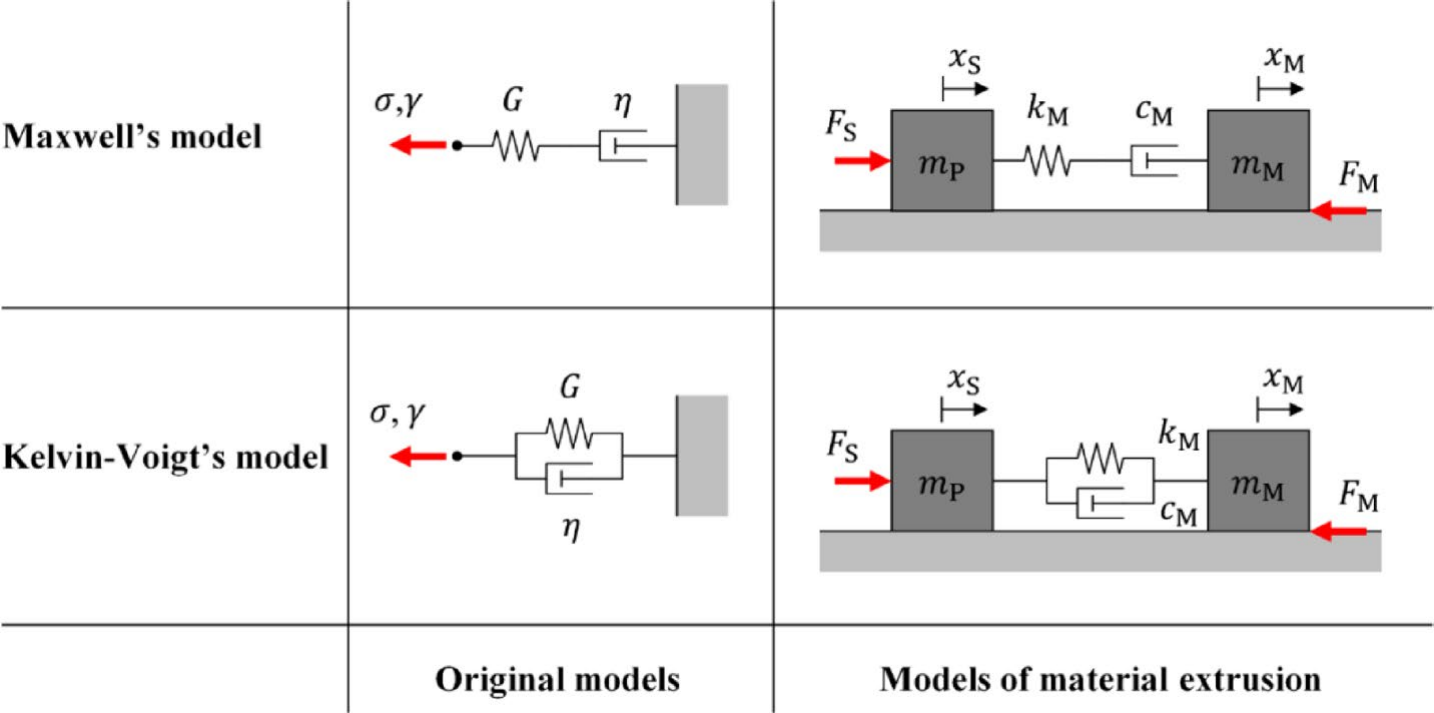
Mechanical analogies of Maxwell’s and Kelvin-Voigt’s viscoelastic models.

## Methodology

### System modeling

#### Material rheology

In rheology, material flow and deformation are studied, investigating how the shear stress of material $\:\sigma\:$ relates to the corresponding strain $\:\gamma\:$. Thus, a material, with a proportional relationship between $\:\sigma\:$ and $\:\gamma\:$ where $\:\sigma\:\left(t\right)=G\left(t\right)\gamma\:\left(t\right)$, is called an elastic material: $\:G$ is recognized as a shear modulus. On the other hand, a material, with a proportional relationship between $\:\sigma\:$ and strain rate $\:\dot{\gamma\:}$ where $\:\sigma\:\left(t\right)=\eta\:\left(t\right)\dot{\gamma\:}\left(t\right)$, is known as a viscous material: $\:\eta\:$ is viscosity. Printing materials for DIW span a wide range of pastes and gels and exhibit both elastic and viscous characteristics. The latter are known as viscoelastic materials. The most extensive models for describing viscoelastic material involve Maxwell’s and Kelvin-Voigt’s models^40^. For instance, Maxwell’s model describes the relaxation of $\:\sigma\:$ over time, under constant $\:\gamma\:$. Kelvin-Voigt’s model explains the creep of $\:\gamma\:$ over time, under invariant $\:\sigma\:$. These models can be visualized having mechanical analogies whereby a spring represents elasticity, and a damper represents viscosity (Fig. 2). Maxwell’s model is depicted by a series of a spring and damper. In contrast, Kelvin-Voigt’s model is illustrated by a parallel set of a spring and damper. It is acknowledged that $\:G$ and $\:\eta\:$ are considerably influenced by material conditions, such as temperature and pressure. For clarity, all variables and parameters used in this study are defined in Table 1.

**Table 1:** Nomenclature of variables and parameters.

Symbols	Descriptions
$\:{A}_{\text{C}}\text{,}{A}_{\text{S}}$	Inner cross-sectional area of cylinder and syringe
$\:{c}_{\text{M}}$	Viscous coefficient of printing material damping and flow resistance
$\:{D}_{\text{C}}\text{,}{D}_{\text{S}}$	Inner diameter of cylinder and syringe
$\:{D}_{\text{W}}$	Screw nominal diameter
$\:{F}_{\text{M}}\text{,}{F}_{\text{V}}$	Printing material flow resistance and internal viscoelasticity force
$\:{F}_{\text{C}}\text{,}{F}_{\text{S}}$	Pneumatic force on pneumatic cylinder and syringe piston
$\:{F}_{\text{W}}$	Screw load
$\:{f}_{\text{T}}\text{,}{f}_{\text{O}}$	Static and viscous components of flow resistance
$\:G$	Printing material shear modulus
$\:{J}_{\text{R}}\text{,}{J}_{\text{W}}$	Moment of inertia of motor and screw
$\:{k}_{\text{M}}$	Printing material spring coefficient
$\:{l}_{\text{C}}\text{,}{l}_{\text{S}}$	Inner axial distance of cylinder and syringe
$\:{m}_{\text{C}}\text{,}{m}_{\text{S}}{\text{,}m}_{\text{D}}$	Air mass in cylinder, syringe, and dead volume
$\:{m}_{\text{P}}\text{,}{m}_{\text{M}}{\text{,}m}_{\text{W}}$	Mass of syringe piston, printing material, and screw carriage
$\:{P}_{\text{a}\text{t}\text{m}}$	Absolute atmospheric pressure
$\:{P}_{\text{C}}\text{,}{P}_{\text{S}}{\text{,}P}_{\text{D}}$	Absolute air pressure in cylinder, syringe, and dead volume
$\:s$	Printing distance
$\:t$	Time
$\:{T}_{\text{C}}\text{,}{T}_{\text{S}}{\text{,}T}_{\text{D}}$	Air temperature in cylinder, syringe, and dead volume
$\:{V}_{\text{C}}\text{,}{V}_{\text{S}}{\text{,}V}_{\text{D}}$	Air volume in cylinder, syringe, and dead volume
$\:w$	Printing linewidth
$\:{x}_{\text{C}}\text{,}{x}_{\text{S}}$	Linear displacement of cylinder and syringe piston
$\:{x}_{\text{M}}\text{,}{x}_{\text{W}}$	Linear displacement of printing material entering nozzle and screw
$\:\alpha\:$	Screw lead angle
$\:\beta\:$	Screw friction angle
$\:\gamma\:$	Shear strain of printing material
$\:\eta\:$	Printing material viscosity
$\:{\theta\:}_{\text{R}}\text{,}{\theta\:}_{\text{W}}$	Angular displacement of motor and screw
$\:\mu\:$	Screw friction coefficient
$\:{\rho\:}_{\text{C}}{\text{,}\rho\:}_{\text{S}}{\text{,}\rho\:}_{\text{D}}$	Air density in cylinder, syringe, and dead volume
$\:{\rho\:}_{\text{M}}$	Printing material density
$\:\sigma\:$	Shear stress of printing material
$\:{\tau\:}_{\text{R}}\text{,}{\tau\:}_{\text{W}}$	Torque of motor and screw

**Fig. 3:**
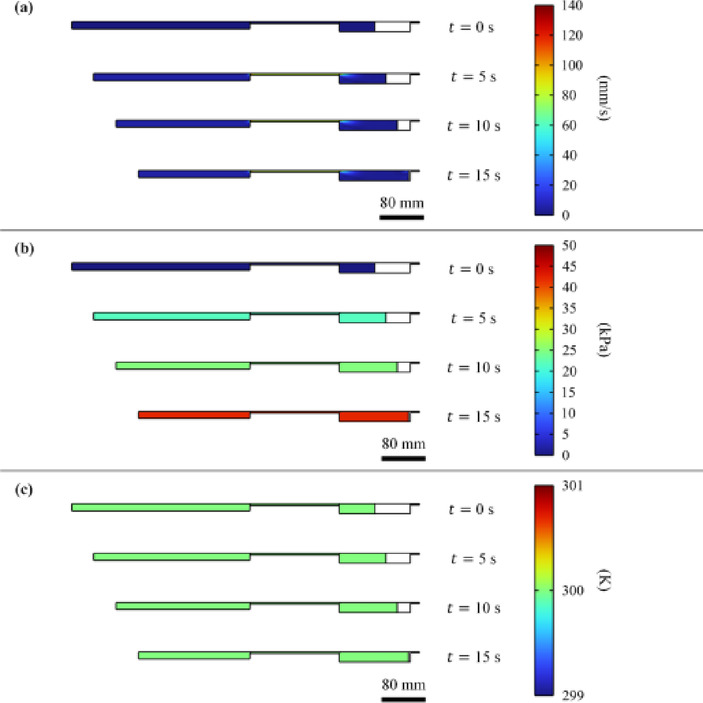
Results of air speed, gauge pressure, and temperature profiles due to simulation of the extrusion of a custom liquid material to determine the transient gradients of air properties in pneumatic transmission: (**a**) Speed profile, (**b**) Gauge pressure profile, and (**c**) Temperature profile.

In this research, Maxwell’s and Kelvin-Voigt’s models are applied to model the behavior of printing material during extrusion. In Fig. 3, a syringe piston is represented by a mass $\:{m}_{\text{P}}$, and a printing material is depicted by a mass $\:{m}_{\text{M}}$. The displacement $\:{x}_{\text{S}}$ denotes the displacement of the piston, and $\:{x}_{\text{M}}$ refers to the material entering the nozzle. Both material elasticity and viscosity are represented by a spring and a damper. The values of the spring constant $\:{k}_{\text{M}}$ and damping coefficient $\:{c}_{\text{M}}$ reflect the amount of $\:G$ and $\:\eta\:$. The piston is propelled by $\:{F}_{\text{S}}$ pushing the printing material, the motion of which is resisted by $\:{F}_{\text{M}}$. Herein, $\:{F}_{\text{M}}$ is modelled as $\:{F}_{\text{M}}\left(t\right)={{f}_{\text{T}}\left(t\right)+c}_{\text{O}}\left(t\right){\dot{x}}_{\text{M}}\left(t\right)$, where $\:{f}_{\text{T}}$ denotes the static component and $\:{c}_{\text{O}}$ is the coefficient associated with the viscous component. Based on material conditions, $\:{k}_{\text{M}}$, $\:{c}_{\text{M}}$, $\:{f}_{\text{T}}$, and $\:{c}_{\text{O}}$ are all seen to vary. Thus, the applied version of Maxwell’s model can be written as:

1 $$\:\left[\begin{array}{c}{\ddot{x}}_{\text{S}}\left(t\right)\\\:{\ddot{x}}_{\text{M}}\left(t\right)\\\:{\dot{F}}_{\text{V}}\left(t\right)\end{array}\right]=\left[\begin{array}{c}\frac{{-{F}_{\text{V}}\left(t\right)+F}_{\text{S}}\left(t\right)}{{m}_{\text{P}}}\\\:\frac{{-{f}_{\text{T}}\left(t\right)-{c}_{\text{O}}\left(t\right){\dot{x}}_{\text{M}}\left(t\right)+F}_{\text{V}}\left(t\right)}{{m}_{\text{M}}\left(t\right)}\\\:{k}_{\text{M}}\left(t\right)\left({\dot{x}}_{\text{S}}\left(t\right)-{\dot{x}}_{\text{M}}\left(t\right)\right)-\frac{{k}_{\text{M}}\left(t\right){F}_{\text{V}}\left(t\right)}{{c}_{\text{M}}\left(t\right)}\end{array}\right]\text{}$$

where $\:{F}_{\text{V}}$ is the internal force caused by the material’s viscoelasticity. Meanwhile, the modified version of Kelvin-Voigt’s model can be expressed as:

2 $$\:\left[\begin{array}{c}{\ddot{x}}_{\text{S}}\left(t\right)\\\:{\ddot{x}}_{\text{M}}\left(t\right)\end{array}\right]=\left[\begin{array}{c}\frac{{-k}_{\text{M}}\left(t\right)\left({x}_{\text{S}}\left(t\right)-{x}_{\text{M}}\left(t\right)\right)-{c}_{\text{M}}\left(t\right)\left({\dot{x}}_{\text{S}}\left(t\right)-{\dot{x}}_{\text{M}}\left(t\right)\right)+{F}_{\text{S}}\left(t\right)}{{m}_{\text{P}}}\\\:\frac{{-{f}_{\text{T}}\left(t\right)-{c}_{\text{O}}\left(t\right){\dot{x}}_{\text{M}}\left(t\right)+k}_{\text{M}}\left(t\right)\left({x}_{\text{S}}\left(t\right)-{x}_{\text{M}}\left(t\right)\right)+{c}_{\text{M}}\left(t\right)\left({\dot{x}}_{\text{S}}\left(t\right)-{\dot{x}}_{\text{M}}\left(t\right)\right)}{{m}_{\text{M}}\left(t\right)}\end{array}\right].$$

Equations (1) and (2) show that $\:{\dot{x}}_{\text{M}}$ is delayed by $\:{\dot{x}}_{\text{S}}$. These equations reveal that the relationship between $\:{\ddot{x}}_{\text{S}}$ and $\:{\ddot{x}}_{\text{M}}$ yields:

3 $$\:{\ddot{x}}_{\text{M}}\left(t\right)=\frac{-{f}_{\text{T}}\left(t\right)-{c}_{\text{O}}\left(t\right){\dot{x}}_{\text{M}}\left(t\right){-m}_{\text{P}}{\ddot{x}}_{\text{S}}\left(t\right)+{F}_{\text{S}}\left(t\right)}{{m}_{\text{M}}\left(t\right)}\text{.}$$

This correlation illustrates that $\:{F}_{\text{S}}\left(t\right)={f}_{\text{T}}\left(t\right)+{c}_{\text{O}}\left(t\right){\dot{x}}_{\text{M}}\left(t\right)$ is at steady state, and $\:{\dot{x}}_{\text{M}}$ ultimately converges with $\:{\dot{x}}_{\text{S}}$. Under pressure control, the extrusion input is equivalent to steps of $\:{F}_{\text{S}}$, whereas in feedrate control, it corresponds to steps of $\:{\dot{x}}_{\text{S}}$. Over time, $\:{m}_{\text{M}}$ decays and material conditions vary. During pressure control, $\:{\dot{x}}_{\text{S}}$ and $\:{\dot{x}}_{\text{M}}$ are likely to diverge from their initial values. Meanwhile, $\:{\dot{x}}_{\text{S}}$ and $\:{\dot{x}}_{\text{M}}$ are found to be more consistent with feedrate control. Nonetheless, a stepper motor can slip when $\:{F}_{\text{M}}$ is excessive, making $\:{\dot{x}}_{\text{S}}$ and $\:{\dot{x}}_{\text{M}}$ unpredictable during the process.

**Table 2:** Parameters and conditions for simulation of the extrusion of a custom liquid material.

Parameters	Values
Simulation time	15 s
Sampling time	100 ms
Mesh size	50–980 μm
$\:{\dot{x}}_{\text{C}}$	5 mm/s
$\:{D}_{\text{C}}$	16 mm
$\:{l}_{\text{C}}$	200 mm
$\:{D}_{\text{S}}$	22.5 mm
$\:{l}_{\text{S}}$	40 mm
$\:{V}_{\text{D}}$	1.256 L
Material density	1.2 Gg/m^3^
Material viscosity	20 Pa·s
Material thermal conductivity	200 mW/(m·K)
Material heat capacity	2 kJ/(kg·K)
Wall thermal conductivity	Perfect insulation
Wall slip condition	Slip
Initial temperature	300 K
Initial pressure	$\:{P}_{\text{a}\text{t}\text{m}}$ (101.325 kPa)

## Pneumatic transmission

Pneumatic transmission is the transmission of power through compressible air. A pneumatic connection is flexible, so the driven side can move with low constraint to the driving side. In this research, the driving side uses a pneumatic cylinder, and the driven side uses a pneumatic syringe. The connection between the cylinder and syringe contains a chamber of dead volume. Navier-Stroke’s Eq.^67^ suggest that during the flow of air, the properties of air (velocity, pressure, and density) are governed by simultaneous nonlinear dynamic relationships^68^. To determine the transient gradients of air properties in pneumatic transmission, we conducted a simulation of liquid extrusion. Simulation includes the studies of laminar flow and heat transfer in a 2D axisymmetric domain. In Table 2, simulation parameters and conditions are demonstrated. In Fig. 3, simulation results reveal that both pressure and temperature gradients are negligible under velocity difference below 140 mm/s. Results also show there is no change in temperature.

When air pressure and temperature are uniform, air mass in the cylinder $\:{m}_{\text{C}}$ is a function of the corresponding pressure $\:{P}_{\text{C}}$, temperature $\:{T}_{\text{C}}$, volume $\:{V}_{\text{C}}$, and density $\:{\rho\:}_{\text{C}}$. Likewise, it is noted that air mass in the syringe $\:{m}_{\text{S}}$ along with dead volume $\:{m}_{\text{D}}$ are functions of their corresponding pressure $\:{P}_{\text{S}}$ and $\:{P}_{\text{D}}$, temperature $\:{T}_{\text{S}}$ and $\:{T}_{\text{D}}$, volume $\:{V}_{\text{S}}$ and $\:{V}_{\text{D}}$, and density $\:{\rho\:}_{\text{D}}$ and $\:{\rho\:}_{\text{S}}$. The mass flow rate of the air in the cylinder, syringe, and dead volume can be expressed as:

4 $$\:\left[\begin{array}{c}{\dot{m}}_{\text{C}}\left(t\right)\\\:{\dot{m}}_{\text{D}}\left(t\right)\\\:{\dot{m}}_{\text{S}}\left(t\right)\end{array}\right]=\left[\begin{array}{c}\frac{\partial\:{m}_{\text{C}}}{\partial\:{P}_{\text{C}}}{\dot{P}}_{\text{C}}\left(t\right)+\frac{\partial\:{m}_{\text{C}}}{\partial\:{T}_{\text{C}}}{\dot{T}}_{\text{C}}\left(t\right)+\frac{\partial\:{m}_{\text{C}}}{\partial\:{V}_{\text{C}}}{\dot{V}}_{\text{C}}\left(t\right)\\\:\frac{\partial\:{m}_{\text{D}}}{\partial\:{P}_{\text{D}}}{\dot{P}}_{\text{D}}\left(t\right)+\frac{\partial\:{m}_{\text{D}}}{\partial\:{T}_{\text{D}}}{\dot{T}}_{\text{D}}\left(t\right)+\frac{\partial\:{m}_{\text{D}}}{\partial\:{V}_{\text{D}}}{\dot{V}}_{\text{D}}\left(t\right)\\\:\frac{\partial\:{m}_{\text{S}}}{\partial\:{P}_{\text{S}}}{\dot{P}}_{\text{S}}\left(t\right)+\frac{\partial\:{m}_{\text{S}}}{\partial\:{T}_{\text{S}}}{\dot{T}}_{\text{S}}\left(t\right)+\frac{\partial\:{m}_{\text{S}}}{\partial\:{V}_{\text{S}}}{\dot{V}}_{\text{S}}\left(t\right)\end{array}\right]\text{.}$$

Because the air is enclosed, $\:{\dot{m}}_{\text{C}}\left(t\right)+{\dot{m}}_{\text{D}}\left(t\right)+{\dot{m}}_{\text{S}}\left(t\right)=0\text{.}$ Other constraints are $\:{\rho\:}_{\text{C}}\left(t\right)={\rho\:}_{\text{D}}\left(t\right)={\rho\:}_{\text{S}}\left(t\right)$,

$\:{P}_{\text{C}}\left(t\right)={P}_{\text{D}}\left(t\right)={P}_{\text{S}}\left(t\right)={F}_{\text{C}}\left(t\right)/{A}_{\text{C}}={F}_{\text{S}}\left(t\right)/{A}_{\text{S}}$ and $\:{\dot{T}}_{\text{C}}\left(t\right)={\dot{T}}_{\text{D}}\left(t\right)={\dot{T}}_{\text{S}}\left(t\right)=0$. Moreover, it is assumed that $\:{\left.\partial\:{m}_{\text{C}}/\:\partial\:{P}_{\text{C}}\right|}_{t}={\rho\:}_{\text{S}}\left(t\right){V}_{\text{C}}\left(t\right)/{P}_{\text{S}}\left(t\right)$, $\:{\left.\partial\:{m}_{\text{D}}/\:\partial\:{P}_{\text{D}}\right|}_{t}={\rho\:}_{\text{S}}\left(t\right){V}_{\text{D}}\left(t\right)/{P}_{\text{S}}\left(t\right)$, and $\:{\left.\partial\:{m}_{\text{S}}/\:\partial\:{P}_{\text{S}}\right|}_{t}={\rho\:}_{\text{S}}\left(t\right){V}_{\text{S}}\left(t\right)/{P}_{\text{S}}\left(t\right)$, while $\:{\left.\partial\:{m}_{\text{C}}/\:\partial\:{V}_{\text{C}}\right|}_{t}={\left.\partial\:{m}_{\text{D}}/\:\partial\:{V}_{\text{D}}\right|}_{t}={\left.\partial\:{m}_{\text{S}}/\:\partial\:{V}_{\text{S}}\right|}_{t}={\rho\:}_{\text{S}}\left(t\right)$. The inner cross-sectional areas of the cylinder and syringe are represented by parameters $\:{A}_{\text{C}}$ and $\:{A}_{\text{S}}$, respectively. The force $\:{F}_{\text{C}}$ denotes the force load on the cylinder piston, and $\:{x}_{\text{C}}$ refers to the displacement of the piston. Combining Eq. (4) along with all constraints allows $\:{\dot{x}}_{\text{C}}$ and $\:{\dot{x}}_{\text{S}}$ to be determined as:

5 $$\:{\dot{x}}_{\text{S}}\left(t\right)=\frac{{A}_{\text{C}}{\dot{x}}_{\text{C}}\left(t\right)}{{A}_{\text{S}}}-\frac{{P}_{\text{S}}\left(0\right)\left({A}_{\text{C}}{l}_{\text{C}}+{V}_{\text{D}}+{A}_{\text{S}}{l}_{\text{S}}\right){\dot{F}}_{\text{S}}\left(t\right)}{{A}_{\text{S}}{A}_{\text{C}}{\left({P}_{\text{a}\text{t}\text{m}}+\frac{{F}_{\text{C}}\left(t\right)}{{A}_{\text{C}}}\right)}^{2}}\text{,}$$

where $\:{P}_{\text{a}\text{t}\text{m}}$ is atmospheric pressure. Parameters $\:{l}_{\text{C}}$ and $\:{l}_{\text{S}}$ are the inner axial distance of the cylinder and syringe. Let $\:{D}_{\text{C}}$ and $\:{D}_{\text{S}}$ be the inner diameter of the cylinder and syringe. Thus, the correlation between $\:{F}_{\text{C}}$ and $\:{F}_{\text{S}}$ can be written as:

6 $$\:{F}_{\text{S}}\left(t\right)=\frac{{D}_{\text{S}}^{2}{F}_{\text{C}}\left(t\right)}{{D}_{\text{C}}^{2}}\text{.}$$

According to $\:{\dot{F}}_{\text{S}}$, Eq. (5) reveals that the response of $\:{\dot{x}}_{\text{S}}$ is delayed by $\:{\dot{x}}_{\text{C}}$. Pressure $\:{P}_{\text{S}}$ is restricted to be positive. Therefore, pneumatic transmission is strongly asymmetric and time delayed. This type of transmission enables the measurement of $\:{P}_{\text{S}}$, so that $\:{F}_{\text{S}}$ and $\:{\dot{F}}_{\text{S}}$ are perceivable over time. On the other hand, the determination of $\:{\dot{x}}_{\text{S}}$ is much more challenging, as it is difficult to be directly quantified, owing to the limited space in the syringe. Therefore, we suggest that air models should be employed to approximate $\:{\dot{l}}_{\text{S}}$, thereby enabling the estimation of $\:{\dot{x}}_{\text{S}}$.

## Linear actuator

A linear actuator consists of two main components: a screw mechanism and a DC brushed motor. A screw mechanism is responsible for transmitting mechanical rotation to translation. The translation side of the screw is coupled with the cylinder piston. Meanwhile, the rotation side of the screw is mounted on the motor rotor. Accordingly, the screw’s linear displacement $\:{x}_{\text{W}}$ and force $\:{F}_{\text{W}}$ can be described as $\:{x}_{\text{W}}\left(t\right)={x}_{\text{C}}\left(t\right)$ and $\:{F}_{\text{W}}\left(t\right)={F}_{\text{C}}\left(t\right)$, respectively. Let $\:{\theta\:}_{\text{W}}$ and $\:{\tau\:}_{\text{W}}$ represent the angular displacement and torque of the screw. The correlation between $\:{x}_{\text{W}}$ and $\:{\theta\:}_{\text{W}}$ is linear:

7 $$\:{x}_{\text{W}}\left(t\right)=\frac{{D}_{\text{W}}\text{tan}\left(\alpha\:\right){\theta\:}_{\text{W}}\left(t\right)}{2}\text{,}$$

where $\:\alpha\:$ is the lead angle of the screw. When the inertia of the ball screw’s shaft and carriage is accounted for in the dynamic derivation, the equation of motion reveals that the relationship between $\:{F}_{\text{W}}$ and $\:{\tau\:}_{\text{W}}$ is under the influence of $\:{\ddot{x}}_{\text{W}}$:

8 $$\:{F}_{\text{W}}\left(t\right)=-\frac{4{J}_{\text{W}}{\ddot{x}}_{\text{W}}\left(t\right)}{{D}_{\text{W}}^{2}\text{tan}\left(\alpha\:+\beta\:\right)\text{tan}\left(\alpha\:\right)}-{m}_{\text{W}}{\ddot{x}}_{\text{W}}\left(t\right)+\frac{2{\tau\:}_{\text{W}}\left(t\right)}{{D}_{\text{W}}\text{tan}\left(\alpha\:+\beta\:\right)}\text{,}$$

where $\:{J}_{\text{W}}$ and $\:{m}_{\text{W}}$ depict the moment of inertia of the screw shaft and the mass of the screw carriage, respectively^63^. The angle $\:\beta\:$ is the friction angle, which is defined based on the screw friction coefficient $\:\mu\:$ as $\:\beta\:=\pm\:{\text{tan}}^{-1}\left(\mu\:\right)\text{}$.

To drive the system, the motor is exploited. As its rotor is coupled with the screw shaft, $\:{\theta\:}_{\text{R}}\left(t\right)={\theta\:}_{\text{W}}\left(t\right)$ and $\:{\tau\:}_{\text{R}}\left(t\right)={\tau\:}_{\text{W}}\left(t\right)$. Thus, $\:{\theta\:}_{\text{R}}$ and $\:{\tau\:}_{\text{R}}$ represent both motor angular displacement and torque. The motor rotor is also equipped with an incremental encoder, so $\:{\theta\:}_{\text{R}}$ is directly measured over time. The time-derivatives of $\:{\theta\:}_{\text{R}}$ can then be determined. Additionally, the equation of motion suggests that $\:{\ddot{\theta\:}}_{\text{R}}\left(t\right)={\tau\:}_{\text{R}}\left(t\right)/{J}_{\text{R}}$ where $\:{J}_{\text{R}}$ is the moment of inertia of the motor rotor.

## System overview

The system model was developed by combining the models of all components. By substituting both displacement and force variables in Eq. (3), $\:{\ddot{x}}_{\text{M}}$ was determined as a function of $\:{\dot{x}}_{\text{M}}$, $\:{\tau\:}_{\text{R}}$, and $\:{\ddot{\tau\:}}_{\text{R}}$, as follows:

9 $$\begin{aligned} \:\ddot{x}_{{\text{M}}} \left( t \right) = & - \frac{{f_{{\text{T}}} \left( t \right) + c_{{\text{O}}} \left( t \right)\dot{x}_{{\text{M}}} \left( t \right)}}{{m_{{\text{M}}} \left( 0 \right) - \frac{{D_{{\text{S}}}^{2} \rho \:_{{\text{M}}} \left( t \right)x_{{\text{M}}} \left( t \right)}}{4}}} + \frac{{2A_{2} D_{{\text{S}}}^{2} \tau \:_{{\text{R}}} \left( t \right)}}{{A_{1} \left( {m_{{\text{M}}} \left( 0 \right) - \frac{{D_{{\text{S}}}^{2} \rho \:_{{\text{M}}} \left( t \right)x_{{\text{M}}} \left( t \right)}}{4}} \right)}} \\ & + \frac{{m_{{\text{P}}} D_{{\text{C}}}^{2} D_{{\text{W}}} {\text{tan}}\left( {\alpha \:} \right)\tau \:_{{\text{R}}} \left( t \right)}}{{2J_{{\text{R}}} D_{{\text{S}}}^{2} \left( {m_{{\text{M}}} \left( 0 \right) - \frac{{D_{{\text{S}}}^{2} \rho \:_{{\text{M}}} \left( t \right)x_{{\text{M}}} \left( t \right)}}{4}} \right)}} \\ & + \frac{{32P_{{\text{S}}} \left( 0 \right)A_{2} \left( {V_{{\text{D}}} + \frac{{l_{{\text{C}}} D_{{\text{C}}}^{2} }}{4} + \frac{{l_{{\text{S}}} D_{{\text{S}}}^{2} }}{4}} \right)\mathop {\tau \:}\limits^{{..}} _{{\text{R}}} \left( t \right)}}{{A_{1} D_{{\text{S}}}^{2} \left( {P_{{{\text{atm}}}} + \frac{{8A_{2} }}{{A_{1} }}} \right)^{2} \left( {m_{{\text{M}}} \left( 0 \right) - \frac{{D_{{\text{S}}}^{2} \rho \:_{{\text{M}}} \left( t \right)x_{{\text{M}}} \left( t \right)}}{4}} \right)\tau \:_{{\text{R}}} \left( t \right)}}{\text{,}} \\ \end{aligned} F$$

where $\:{A}_{1}$, $\:{A}_{2}$, and $\:{A}_{3}$ are arbitrary constant parameters. The parameters are defined as $\:{A}_{1}={D}_{\text{C}}^{2}{D}_{\text{W}}\text{tan}\left(\alpha\:+\beta\:\right)$, $\:{A}_{2}=1-{A}_{3}/{J}_{\text{R}}$, and $\:{A}_{3}={m}_{\text{W}}\text{tan}\left(\alpha\:+\beta\:\right)\text{tan}\left(\alpha\:\right){D}_{\text{W}}^{2}/4+{J}_{\text{W}}$. This model demonstrates that the system is second-order nonlinear with multiple inputs and time-varying parameters.

### System analysis

To analyze the system, assumptions were applied to the nonlinear model. The assumptions comprised: $\:{\ddot{\tau\:}}_{\text{R}}\left(t\right)=0$, $\:{m}_{\text{M}}\left(t\right)={m}_{\text{M}}\left(0\right)$, $\:{f}_{\text{T}}\left(t\right)={f}_{\text{T}}$, and $\:{c}_{\text{O}}\left(t\right)={c}_{\text{O}}$. Using these assumptions, the model was linearized. Therefore, analysis methods for linear time-invariant systems can be applied. A linear model was developed:

10 $$\:{\ddot{x}}_{\text{M}}\left(t\right)=-\frac{{c}_{\text{O}}{\dot{x}}_{\text{M}}\left(t\right)+{f}_{\text{T}}}{{m}_{\text{M}}\left(0\right)}+\frac{{m}_{\text{P}}{{D}_{\text{W}}D}_{\text{C}}^{2}{\tau\:}_{\text{R}}\left(t\right)}{2{m}_{\text{M}}\left(0\right){J}_{\text{R}}{D}_{\text{S}}^{2}}+\frac{2{D}_{\text{S}}^{2}\left(1-\frac{{m}_{\text{W}}{\text{tan}\left(\alpha\:\right)\text{tan}\left(\alpha\:+\beta\:\right)D}_{\text{W}}^{2}+{4J}_{\text{W}}}{4{J}_{\text{R}}}\right){\tau\:}_{\text{R}}\left(t\right)}{{m}_{\text{M}}\left(0\right)\text{tan}\left(\alpha\:+\beta\:\right){{D}_{\text{W}}D}_{\text{C}}^{2}}$$

The poles of the linear model were determined as 0 and $\:-{c}_{\text{O}}/{m}_{\text{M}}\left(0\right)$. Thus, it is revealed that $\:{x}_{\text{M}}$ is marginally stable, and $\:{\dot{x}}_{\text{M}}$ is asymptotically stable. With the input of $\:{\tau\:}_{\text{R}}$, the linear model also implies that the system is controllable. Nevertheless, $\:{m}_{\text{M}}$, $\:{f}_{\text{T}}$, and $\:{c}_{\text{O}}$ are observed to fluctuate in practice and significantly diverge from their original values. The actual system is literally much more intricate than the linear model. To maintain the stability of the system and provide steady $\:{\dot{x}}_{\text{M}}$, there is no conclusion for optimum solutions.

## Stationary extrusion

**Fig. 4:**
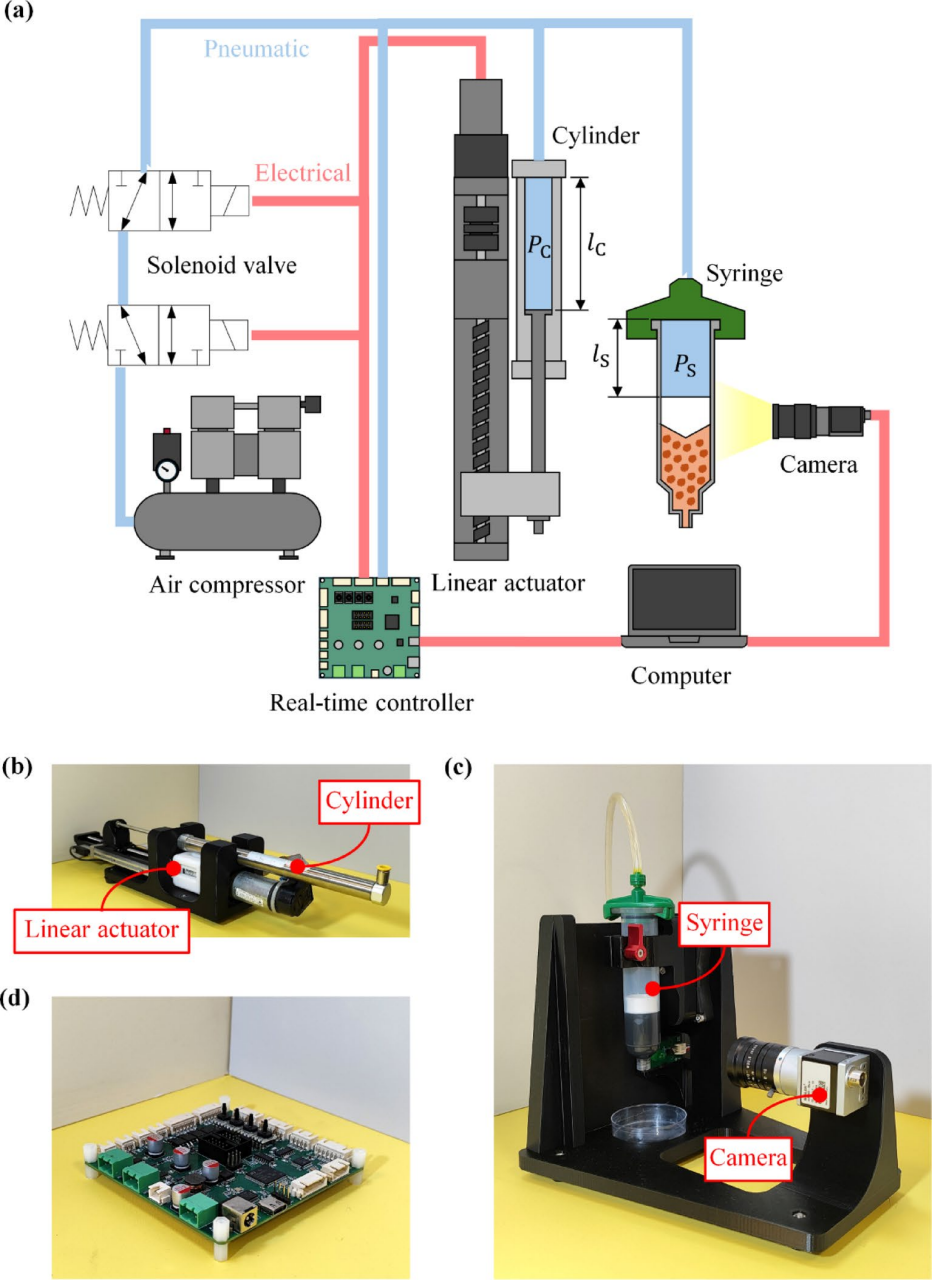
Experimental apparatus for the stationary extrusion of gelatin: (**a**) Architecture of the system, (**b**) Pneumatic cylinder and linear actuator, (**c**) Pneumatic syringe and camera, and (d) Real-time controller.

The proposed methodology was evaluated through a series of experiments. One of them was an investigation into $\:{\dot{l}}_{\text{S}}$ during material extrusion while the printhead was stationary. In Fig. 4a, the architecture of the investigation is shown. The pneumatic cylinder is an industrial type (SMC CDJ2B16-200Z-B). In Fig. 4b, the piston was motorized via a linear stage. The linear stage is driven by a brushed DC motor (Maxon A-max 254609) with a gear transmission of 18:1. Motor rotation was tracked using an incremental encoder (Avago HEDL-5540), and transmitted into linear motion, employing a ball-screw rail (Misumi LX2001-B1-F-200). The cylinder was pneumatically connected to a 30-ml plastic pneumatic syringe, which was fixed in a vertical position (Fig. 4c). The inner diameter of the syringe at its barrel was 22.5 mm. At the outlet of the syringe, a 14-gauge taper dispensing nozzle was attached. The inner diameter of the nozzle at its vent was 1.6 mm. Under the nozzle, a petri dish was placed to hold the extruded material. For pressure preloading, the pneumatic connection between the cylinder and syringe was connected to an air compressor through a pair of bi-directional solenoid valves (SMC VT307). The piston of the syringe was captured using a monochrome USB camera (Basler acA1300-30 μm) with a 6-mm focal lens (Ricoh FL-HC0614-2 M). Both the syringe and camera were fitted to the same chassis. To harmonize the extrusion system and camera, a light emitting diode (LED) module was fixed next to the syringe. Intermediate components used in these modules and their chassis were all 3D-printed.

Once assembled, the printhead weighed 110 g without material loading, and its bounding box was 40 × 65 × 140 cm. This design was compared to our previous printhead configuration, which weighed 630 g and measured 50 × 85 × 230 mm; both systems have comparable capabilities in actuation and sensing extrusion parameters^62^. As a result, the current design is much lighter and smaller.

Meanwhile, the reservoir capacity of the printhead in this study is three times larger, with volumes of 30 ml and 10 ml, respectively.

In this hardware setup, noticeable energy losses occur during the preloading of $\:{P}_{\text{S}}$, particularly when the solenoid valves switch states. To initiate preloading, the solenoid valves first connect the air compressor to the pneumatic cylinder, allowing pressurized air to flow into the air chamber until the pressure stabilizes. Subsequently, the valves are actuated to isolate the chamber. During this transition, however, a portion of the preloaded air is leaked to the atmosphere due to the valve switching process, resulting in a drop in the initial value of $\:{P}_{\text{S}}$. Therefore, this air leakage is the primary source of energy loss in the operation of the extrusion system.

In Fig. 4d, a custom real-time controller is displayed. The entire system is under this controller. Herein, a motor driver, encoder counter, pressure sensor, and two real-time microcontrollers are integrated. The microcontrollers are 32-bit ARM Cortex-M types, and can communicate with each other using SPI protocol. For the connection with peripherals, one microcontroller (STM32G4) was employed, and executed with a real-time firmware running at 10 kHz. The other microcontroller (STM32H7) is responsible for signal processing and system control, running at 250 Hz in interactively conjunction with a laptop computer. The pressure sensor is a miniature type with an operating gauge pressure between 0 and 1.034 MPa (Honeywell ABPDANV150PGSA3). The motor driver is also an on-board type with 12-A maximum current (Texas Instruments DRV8243).

Further, the printing material was gelatin (INS 428) with a concentration of 4.76% w/w. The independent variable was $\:{\dot{l}}_{\text{C}}$, with target values of − 200, − 400, − 600, and − 800 μm/s. The negative values of $\:{\dot{l}}_{\text{C}}$ represent the compression of the air chamber inside the pneumatic cylinder. The experiment was conducted at room temperature (25 °C). At this temperature, the material was in the sol-gel transition phase. First, the original value of $\:{l}_{\text{S}}$ was measured and recorded. The initial condition of $\:{P}_{\text{S}}$ was designated to be 110.15 kPa. To minimize the error of the preload mechanism, $\:{\dot{l}}_{\text{C}}$ was driven at − 6 mm/s until $\:{P}_{\text{S}}$ reached the value. Afterwards, extrusion started and took 60 s. The system was then frozen for 120 s, before the pneumatic connection being neutralized.

During each test, a video capturing the piston of the syringe was recorded: the framerate was 25 Hz. The video was later processed to extract the resulting values of $\:{\dot{x}}_{\text{S}}$. All video frames were cropped, and a feature of the piston in the first frame was then manually extracted. Template matching using fast normalized cross-correlation was implemented to track the extracted feature in all other video frames^69^. The displacement of $\:{x}_{\text{S}}$ was calculated by comparing the piston position from all frames to that of the initial frame. The size of the piston was utilized for unit conversion. For noise elimination, a cubic spline curve fitting was applied to the acquired set of $\:{x}_{\text{S}}$. Finally, $\:{x}_{\text{S}}$ was converted into $\:{l}_{\text{S}}$.

To develop an air model, it is assumed that the product of the total air volume and $\:{P}_{\text{S}}$ remains constant over the period of extrusion and freeze. The dead volume $\:{V}_{\text{D}}$ was investigated and found to be 13.38 cm^3^ while $\:{V}_{\text{C}}$ was instantaneously measured through $\:{l}_{\text{C}}$. Under this assumption, the volume $\:{V}_{\text{S}}$ can be analytically computed, enabling the estimation of $\:{l}_{\text{S}}$. To validate the estimation results, the predicted and measured values of $\:{l}_{\text{S}}$ were compared. Several statistical indicators, including relative root mean square error (rRMSE), coefficients of determination (R^2^, and p-value, were used for this purpose. Specifically, rRMSE, defined as the ratio of RMSE to the total change in the measured values of $\:{l}_{\text{S}}$, quantifies the magnitude of the estimation error relative to the scale of $\:{l}_{\text{S}}$ values. The proportion of variance in the empirical measurements of $\:{l}_{\text{S}}$ explained by the proposed estimation model is represented by R^2^while p-value reflects the statistical significance of this relationship by testing the null hypothesis.

**Fig. 5:**
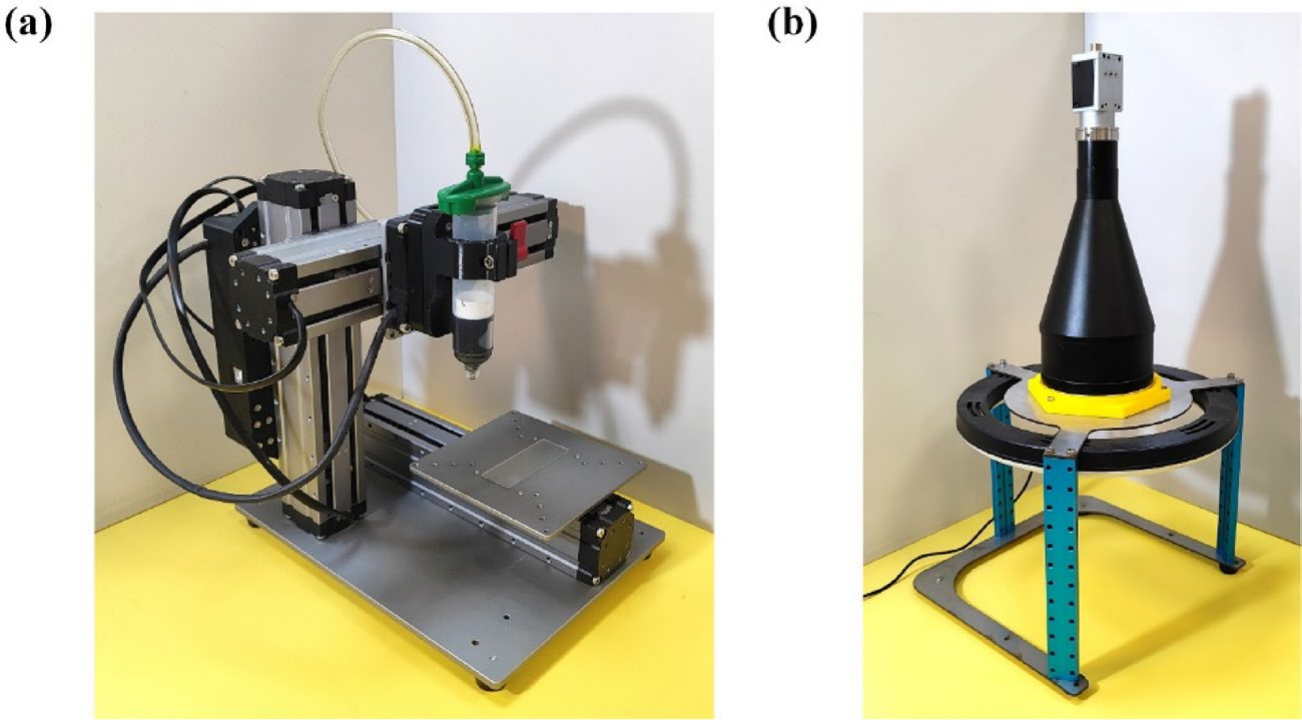
Experimental apparatus for the line printing of gelatin: (**a**) Pneumatic syringe equipped with a cartesian manipulator, and (**b**) Camera with telecentric lens.

## Line printing

In another experiment, the linewidths of printed lines $\:w$ were investigated. In Fig. 5a, the pneumatic syringe was equipped with a cartesian manipulator. Both the extrusion system and manipulator were used to deposit the same material as in the previous experiment on glass slides. There were two printing conditions: $\:{\dot{l}}_{\text{C}}$ of − 200, − 300, and − 400 μm/s with a printing speed of 2 mm/s. Nozzle height (the gap between the nozzle tip and glass slide) was 800 μm, and preload of $\:{P}_{\text{S}}$ was 10 kPa. This experiment was also conducted at room temperature (25 °C). The printed lines were 120 mm in total, and captured using a color USB camera (Basler acA5472-17uc) with a telecentric lens (Fig. 5b). Each image was rotated, so the printed line aligned with the horizontal axis of the resulting image. To analyze $\:w$ values over the final 60 mm of the line, the image was cropped. By sequentially grayscaling and binarizing the cropped image, the printed line was extracted from the background. Afterwards, edge detection was applied to the extracted region^70^. Herein, $\:w$ was defined as the vertical distance between the farthest edge points at each horizontal position. Therefore, along the printing distance $\:s$, the values of $\:w$ were determined. For statistical evaluation of the $\:w$ results, linear regression was subsequently applied. The regression coefficients describe the trends of $\:w$ over $\:s$, while root mean square error (RMSE) quantifies the variation in $\:w$. The goodness of fit between the regression trend and the observed data was assessed through R^2^and p-value was utilized to test the null hypothesis regarding the regression model.

### High-temperature printing

Pneumatic extrusion is extensively employed for dispensing biomaterials at high temperature. Well-known examples of such materials are polycaprolactone (PCL) and polyetheretherketone (PEEK) with pressure-vulnerable additives, such as carbon-based and metal nanomaterials. For high-temperature printing, pneumatic syringes are typically made of metal and operate without an internal piston. When the printing material is nearly depleted, compressed air is likely to infiltrate the remaining material and leak through the nozzle. This phenomenon causes massive material spills on the printed output. Numerous droplets of spilled material are uncontrollably splashed, compromising print quality and increasing the risk of printing failure. Because metal syringes are nontransparent, it is impossible to acknowledge the level of printing material inside. Given this physical constraint, the mentioned phenomenon cannot be avoided systematically. By implementing this architecture, the air leakage is intrinsically observable from the sudden drop of $\:{P}_{\text{S}}$. This capability can improve the quality of printing results significantly.

Herein, 2 g of polypropylene was loaded into a stainless-steel syringe, and extruded at 160 °C without a piston, using both the traditional and proposed pneumatic methods. Extrusion was carried out using a brass nozzle with an outlet diameter of 800 μm, and extrusion time was set to 45 s. To collect the extruded material, a tall shot glass was placed under the nozzle. For the conventional method, $\:{P}_{\text{S}}$ was controlled with a manual pressure regulator at 150 kPa. Meanwhile, in the proposed method, both $\:{\dot{l}}_{\text{C}}$ and the initial level of $\:{P}_{\text{S}}$ were assigned values of − 1 mm/s, and 40 kPa, respectively. Once $\:{P}_{\text{S}}$ dropped below the threshold of 2 kPa, the pressurized air was released and returned to atmospheric pressure. The extruded results were captured using a color USB camera (Raspberry Pi High Quality Camera V1.0) with a telecentric lens (Edmund Optics SilverTL 56675), enabling the observation and comparison of material spills.

## Results and discussion

### Extrusion feedrate and pressure

**Fig. 6:**
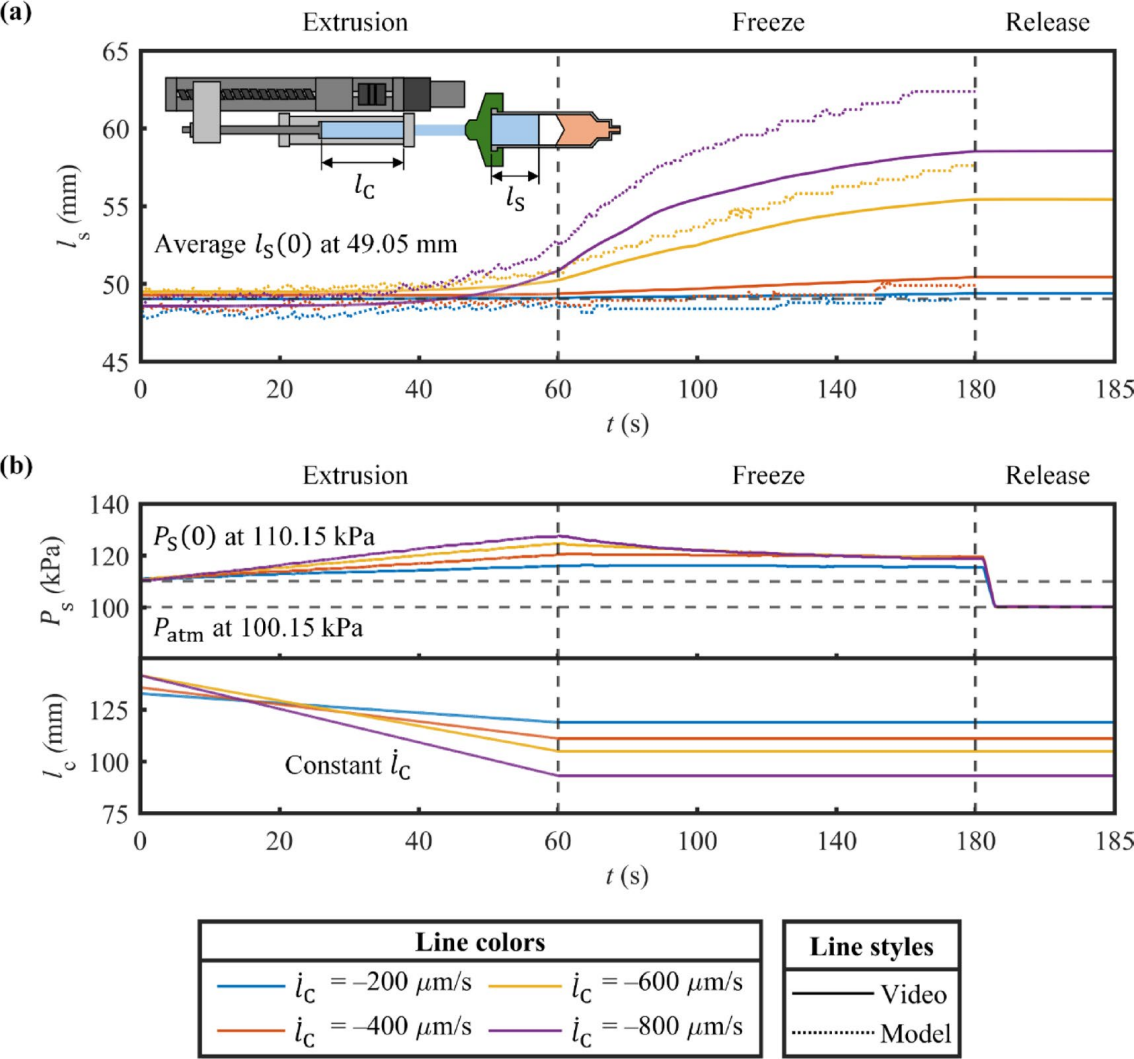
Results of the stationary extrusion of gelatin: (**a**) Inner axial distance of pneumatic syringe $\:{l}_{\text{S}}$, and (**b**) Extrusion pressure $\:{P}_{\text{S}}$ and inner axial distance of pneumatic cylinder $\:{l}_{\text{C}}$.

In Fig. 6a, during stationary extrusion, values of $\:{l}_{\text{S}}$ were successfully collected via video processing and estimation. Using LED, the results of $\:{l}_{\text{S}}$ were synchronized. Under $\:{\dot{l}}_{\text{C}}$ of − 200 and − 400 μm/s, $\:{l}_{\text{S}}$ linearly increased during the extrusion period, and continued to rise at the same rate throughout the freezing period. On the other hand, when $\:{\dot{l}}_{\text{C}}$ was − 600 and − 800 μm/s, the results of $\:{l}_{\text{S}}$ escalated exponentially over time in the extrusion phase, and grew with decaying rates in the freezing phase. Once the pneumatic connection was opened, $\:{l}_{\text{S}}$ remained constant. During the decline of $\:{\dot{l}}_{\text{C}}$, it was obvious that $\:{\dot{l}}_{\text{S}}$ expanded to an increasing degree. As for the aspect of $\:{P}_{\text{S}}$, it was effectively measured in real time during the whole process (Fig. 6b). The growth of $\:{P}_{\text{S}}$ was found consistent and linear throughout extrusion. When the operation of the system was halted, $\:{P}_{\text{S}}$ exponentially declined, especially at higher speeds of $\:{\dot{l}}_{\text{C}}$ (–600 and − 800 μm/s). Under such $\:{\dot{l}}_{\text{C}}$ values, the discovered delays in $\:{\dot{l}}_{\text{S}}$ reveal that it became practically unstable, corresponding to air compressibility. Therefore, for realistic printing, the magnitude of $\:{\dot{l}}_{\text{C}}$ must remain below a certain threshold, which currently needs to be identified with calibration. These results highlight that the proposed system can perform flexible material extrusion with both feedrate and pressure control.

In Fig. 6a, the estimation of $\:{l}_{\text{S}}$ was accomplished. For all $\:{\dot{l}}_{\text{C}}$ values, the estimation results consistently yielded outcomes similar to the experimental results throughout the entire process. As shown in Table 3, statistical evaluation of the estimation proved to be successful. It is noted that rRMSE peaked at 2.051 when $\:{\dot{l}}_{\text{C}}$ was − 200 μm/s. As $\:{\dot{l}}_{\text{C}}$ speed increased, rRMSE declined to 0.562 at $\:{\dot{l}}_{\text{C}}$ of − 400 μm/s, reaching its minimum at 0.205 at $\:{\dot{l}}_{\text{C}}$ of − 600 μm/s before sightly rising to 0.274 at $\:{\dot{l}}_{\text{C}}$ of − 800 μm/s. These rRMSE values indicate that estimation accuracy improves with increasing magnitude of $\:{\dot{l}}_{\text{C}}$. The higher rRMES values at lower speeds of $\:{\dot{l}}_{\text{C}}$ may be attributed to poor signal-to-noise ratio, caused by the limited accuracy and resolution of the pressure sensor. The modest increase in rRMSE at $\:{\dot{l}}_{\text{C}}$ of − 800 μm/s suggests that high-speed operation introduces additional error, possibly due to control latency or inertia effects. Furthermore, a monotonic increase in R^2^ is observed with increasing $\:{\dot{l}}_{\text{C}}$ magnitude, from 0.703 at $\:{\dot{l}}_{\text{C}}$ of − 200 μm/s to 0.999 at $\:{\dot{l}}_{\text{C}}$ of − 800 μm/s. This trend indicates a progressively strong correlation between the computed and measured values as the speed of $\:{\:\dot{l}}_{\text{C}}$ increases. In addition, p-value remains zero across all $\:{\:\dot{l}}_{\text{C}}$ values, indicating that the relationship between the estimated and actual values is statistically significant throughout the testing range. These indicators strongly support the validity of the proposed estimation method.

Future developments in pressure sensing technologies, specifically tailored for this application, are expected to minimize rRMSE at lower speeds of $\:{\:\dot{l}}_{\text{C}}$, and expand the effective operating range of this estimation technique. With reliable estimation of $\:{l}_{\text{S}}$, real-time compensation for $\:{\dot{l}}_{\text{S}}$ is feasible, alongside intrinsically continuous visibility of $\:{P}_{\text{S}}$. Accordingly, the architecture presented has promising potential to significantly advance the impact to DIW process.

**Table 3:** Statistical evaluation of the Estimation of inner axial distance of pneumatic syringe $\:{l}_{\text{S}}$.

$\:{\dot{\varvec{l}}}_{\text{C}}$ (µm/s)	rRSME	*R* ^2^	*P*-value
–200	2.051	0.703	0
–400	0.562	0.864	0
–600	0.205	0.996	0
–800	0.274	0.999	0

### Printing linewidth

**Fig. 7:**
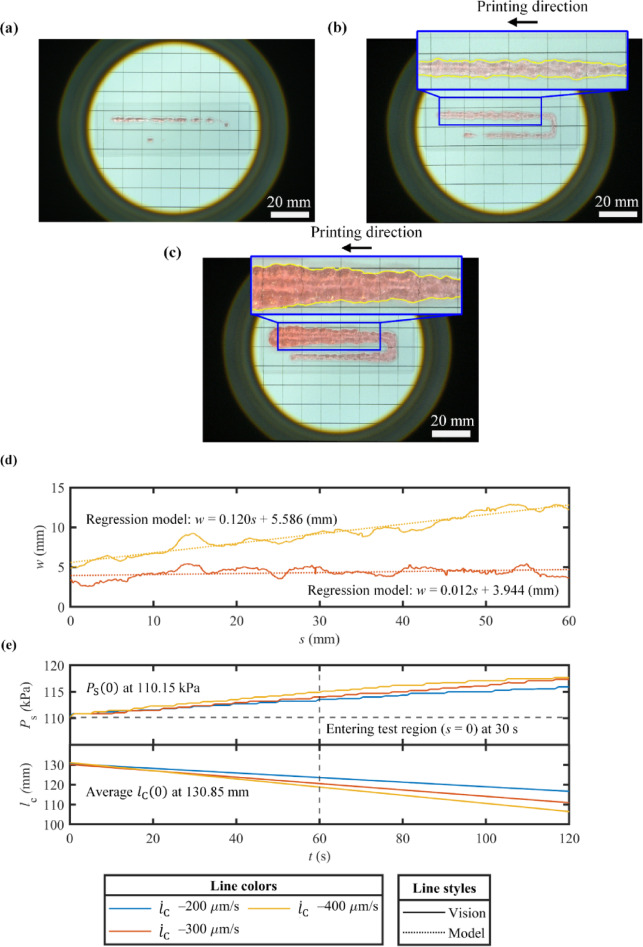
Results of line printing of gelatin: (**a**) Printed line under a feedrate $\:{\dot{l}}_{\text{C}}$ of − 200 μm/s, (**b**) Printed line under a feedrate $\:{\dot{l}}_{\text{C}}$ of − 300 μm/s, (**c**) Printed line under a feedrate $\:{\dot{l}}_{\text{C}}$ of − 400 μm/s, (**d**) Extracted linewidth $\:w$, and (**e**) Extrusion pressure $\:{P}_{\text{S}}$ and inner axial distance of pneumatic cylinder $\:{l}_{\text{C}}$. Results of line printing of gelatin: (**a**) Printed line under a feedrate $\:{\dot{l}}_{\text{C}}$ of − 200 μm/s, (**b**) Printed line under a feedrate $\:{\dot{l}}_{\text{C}}$ of − 300 μm/s, (**c**) Printed line under a feedrate $\:{\dot{l}}_{\text{C}}$ of − 400 μm/s, (**d**) Extracted linewidth $\:w$, and (**e**) Extrusion pressure $\:{P}_{\text{S}}$ and inner axial distance of pneumatic cylinder $\:{l}_{\text{C}}$.

In Fig. 7a, the printing result under $\:{\dot{l}}_{\text{C}}$ of − 200 μm/s is obtained. At this speed, the system failed to provide sufficient material flowrate to construct and maintain a continuous line. As a result, linewidth analysis was not proceeded for this case. In Fig. 7b and c, two printing results ($\:{\dot{l}}_{\text{C}}$ of − 300 and − 400 μm/s) are portrayed. It is seen that extraction of the printed lines was achieved, enabling the detection of their edges. In Fig. 7c, $\:w$ values were measured. In Fig. 7d, under $\:{\dot{l}}_{\text{C}}$ of − 300 μm/s, $\:w$ was steady around 4 mm with some fluctuations. In contrast, when $\:{\dot{l}}_{\text{C}}$ was − 400 μm/s, $\:w$ became unstable, rising from approximately 4 to 12 mm over increases in $\:s$. Similar oscillations in $\:w$ also appear in this condition. The variation in $\:w$ indicates the fact that the printing material was under phase transition between the solution and gel.

In Table 4, statistical evaluation of the resulting $\:w$ under $\:{\dot{l}}_{\text{C}}$ of − 300 and − 400 μm/s is presented. The mean value of $\:w$ was found to be 4.32 mm at $\:{\dot{l}}_{\text{C}}$ of − 300 μm/s. The regression coefficient of 0.012 indicates that $\:w$ remained relatively stable over $\:s$. RMSE of 0.571 reflects the moderate fluctuations in $\:w$, while R^2^ and p-value were reported to be 0.126 and 0, respectively. Considering R^2^the rising trend of $\:w$ over $\:s$ was not statistically significant, indicating that $\:w$ was independent of changes in $\:s$. The null hypothesis over the regression model is denied by the p-value. At $\:{\dot{l}}_{\text{C}}$ of − 400 μm/s, the mean $\:w$ value was at 9.198 mm. The regression coefficient of 0.120 displays a remarkable growing trend in $\:w$, emphasizing that $\:w$ was unstable over $\:s$. RMSE was seen to be 0.566, showing the similar fluctuations between two printing conditions. However, R^2^ of 0.931 and a p-value of 0 confirm that the increase in $\:w$ is both systematic and statistically significant.

In Fig. 7e, the corresponding data of $\:{P}_{\text{S}}$ and $\:{l}_{\text{C}}$ for all printing conditions is illustrated. At $\:{\dot{l}}_{\text{C}}$ of − 200 μm/s, $\:{P}_{\text{S}}$ exhibited an increase from around 110 to 115 kPa. Therefore, the failure of gelatin printing under this printing condition implies that constant or regulated values of $\:{P}_{\text{S}}$ alone are insufficient to ensure the formation of continuous printed lines with stable $\:w$ values. Furthermore, the presented regression trends emphasize that the proposed extrusion technique requires a narrow band of appropriate $\:{\dot{l}}_{\text{C}}$ values to achieve consistent $\:w$ within a specific printing condition. Hence, calibration for optimum values of $\:{\dot{l}}_{\text{C}}$ is still essential for practical implementation.

**Table 4:** Statistical evaluation of the linewidth $\:w$ over printing distance $\:s$ in line printing of gelatin.

$\:{\dot{l}}_{\text{C}}$ (µm/s)	Mean (mm)	Regression model	RMSE (mm)	*R* ^2^	*P*-value
–300	4.319	0.012$\:s$+3.944	0.571	0.126	0
–400	9.198	0.120$\:s$+5.586	0.566	0.931	0

Although 4.76% w/w gelatin was the only material tested in the experiments, this kind of material is extensively utilized in bioprinting. During the transition phase, the gelatin explicitly exhibits heterogeneity, and illustrates extraordinary sensitivity to temperature change. Consequently, the insights obtained from the experimental outcomes can be also applicable to other types of biomaterials.

### Detection of material depletion

**Fig. 8:**
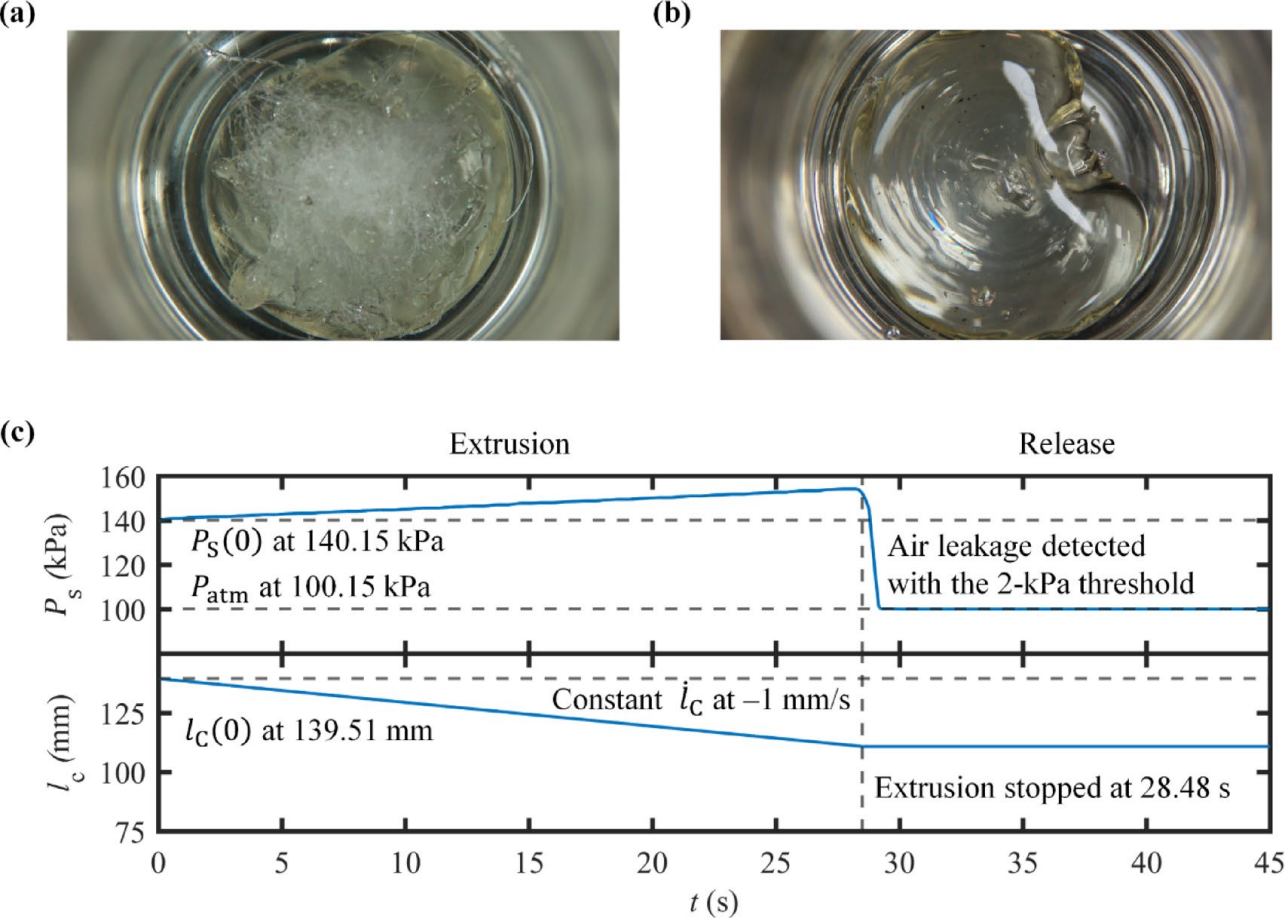
Results of air-leakage detection during the extrusion of polypropylene: (**a**) Extruded material by conventional pneumatic extrusion, and (**b**) Extruded material by flexibly actuated piston-driven pneumatic extrusion with intrinsic air-leakage detection, and (**c**) Extrusion pressure $\:{P}_{\text{S}}$ and inner axial distance of pneumatic cylinder $\:{l}_{\text{C}}$.

The results of traditional pneumatic extrusion process were obtained. During the extrusion process, air leakage was obviously noticeable, leading to overwhelming spills of the printing material. In Fig. 8a, countless opaque material blobs over the top surface of the extruded material are demonstrated. The dispersion of blobs across the entire glass surface highlights the material’s unpredictable spray directions. However, this phenomenon could not be recognized by the system. Although the air leakage was observed in clear vision, no significant change in $\:{P}_{\text{S}}$ was detectable. The piston-driven pneumatic architecture provided a successful result (Fig. 8b). In this case, slight air leakage was perceived, before extrusion was terminated. This termination prevented a massive splash of material on the printed surface. Undesirable droplets of the printing material were remarkably eliminated. The captured image of the result demonstrates its transparency, indicating the disappearance of the droplets. In Fig. 8c, the value of $\:{P}_{\text{S}}$ was recognized in real-time by the system, and linearly inclined over the process. When air started to bleed through the nozzle, the decrease of $\:{P}_{\text{S}}$ began. Once $\:{P}_{\text{S}}$ dropped below the 2-kPa threshold, specifically at 28.48 s, the condition for extrusion stoppage was met. This trigger caused the system to immediately halt $\:{\dot{l}}_{\text{C}}$, thus the compressed air was released into the atmosphere. The comparison between these two results illustrates how printing quality can be enhanced by this technique in practice. While hydrogels typically operate at temperatures below 45 °C, some bioprinting materials, such as PCL and PEEK, require high temperature for extrusion. Such materials are commonly used alongside hydrogels in bioengineering, particularly in bone and cartilage tissue engineering and implantation. Hence, the demonstrated capability of the extrusion method presented is highly advantageous for these applications, advancing bioprinting toward real-world implementation.

## Conclusion

This study introduces a flexibly actuated pneumatic extrusion system capable of real-time extrusion pressure sensing for direct ink writing. By replacing rigid mechanical transmission with pneumatic actuation, the system enables both pressure and feedrate control, supported by an accurate air model. Experimental results validate system suitability for heterogeneous, pressure-sensitive biomaterials, with gelatin lines exhibiting a mean linewidth of 4.32 mm and an increase ratio of 0.012 over printing distance. These findings advance material extrusion in additive manufacturing, particularly for bioprinting and related applications. Future work should focus on enhancing pressure sensing and optimizing transient flow dynamics to improve feedrate estimation and performance in time-sensitive DIW tasks.

## Data Availability

The data supporting the findings of this study are openly available at the following GitHub repository ( [https://github.com/schaiwuth/flexible-diw-pneumatic-extrusion](https:/github.com/schaiwuth/flexible-diw-pneumatic-extrusion) ).
